# The evolving role of physiotherapists in pre-employment screening for workplace injury prevention: are functional capacity evaluations the answer?

**DOI:** 10.1179/1743288X13Y.0000000101

**Published:** 2013-10

**Authors:** Jennifer Legge

**Affiliations:** The University of Queensland, Australia

**Keywords:** Functional capacity evaluation, Musculoskeletal disorders, Physiotherapy, Pre-employment screening, Workplace injuries

## Abstract

**Background:**

Musculoskeletal injuries account for the largest proportion of workplace injuries. In an attempt to predict, and subsequently manage, the risk of sprains and strains in the workplace, employers are turning to pre-employment screening. Functional capacity evaluations (FCEs) are increasing in popularity as a tool for pre-employment screening despite limited published evidence for their validity in healthy working populations.

**Objectives:**

This narrative review will present an overview of the state of the evidence for pre-employment functional testing, propose a framework for decision-making to determine the suitability of assessment tools, and discuss the role and potential ethical challenges for physiotherapists conducting pre-employment functional testing.

**Major Findings:**

Much of the evidence surrounding the validity of functional testing is in the context of the injured worker and prediction of return to work. In healthy populations, FCE components, such as aerobic fitness and manual handling activities, have demonstrated predictability of workplace injury in a small number of studies. This predictability improves when workers' performance is compared with the job demands. This job-specific approach is also required to meet anti-discrimination requirements. There are a number of practical limitations to functional testing, although these are not limited to the pre-employment domain. Physiotherapists need to have a clear understanding of the legal requirements and potential ethical challenges that they may face when conducting pre-employment functional assessments (PEFAs).

**Conclusions:**

Further research is needed into the efficacy of pre-employment testing for workplace injury prevention. Physiotherapists and PEFAs are just one part of a holistic approach to workplace injury prevention.

## Background

There is a strong focus in almost all developed countries, particularly over the last few decades, to achieve a zero rate of injury in the workplace. This is driven by a combination of legislative compliance, the altruistic concerns for employee welfare, as well as the competitive advantage from improved productivity and the commercial realities of rising injury costs, litigation, and criminal prosecution.

### The problem of workplace injuries

Despite this focus, whilst the numbers of injuries are decreasing the costs continue to rise.^1^ A review of recent publications from international representative bodies^2^–^5^ reveals similar trends across jurisdictions: workplace injury rates are decreasing but costs are increasing; musculoskeletal injuries account for the biggest proportion of non-fatal injury types; ‘body stressing’ is the most common mechanism of injury for workers involved in manual work; and the back is the most commonly injured body part of workers requiring time off work.

Workplace injuries have numerous physical, psychological, financial, and social impacts on the injured workers, the employer and their co-workers, and the worker’s family and community. From the perspective of the worker, this is about the value of their loss of income, leisure time, and enjoyment. From the perspective of the employer, it is about the costs including productivity loss, recruitment and retraining costs, fines, and penalties. And from the community’s perspective, it is about the lost value of contribution from the potential future income generation.^6^

The underlying premise of workplace health and safety legislation in industrialized nations, such as the Queensland Work Health and Safety Act 2011,^7^ is a business’ primary duty of care of ensuring so far as reasonably practicable, the health and safety of workers, and other persons who are at work. In relation to manual handling injuries, this includes items such as the provision of a safe workplace, equipment, and handling of structures, provision of training and instruction, and monitoring the health of workers for the purposes of preventing injury.

Workplace injury prevention strategies typically follow a risk management approach such as that described in Australia’s Hazardous Manual Tasks Code of Practice 2011^8^ including identifying hazardous manual tasks, assessing the risks, implementing control measures in accordance with the hierarchy of control, and then reviewing the effectiveness of those control measures.

In relation to manual tasks, these are typically achieved by modifying tasks and equipment in an effort to match the task to the human. Sometimes, due to technical or cost considerations, this approach becomes impractical and the shift then changes to matching the worker to the task. A combination of both matching tasks to workers and workers to tasks is also practised.^9^

The cause of workplace musculoskeletal injuries is multifactorial, including factors such as the workers’ individual, physical, and psychological characteristics, the physical environment, processes, and equipment, and organizational and psychosocial factors.^10^,^11^ This is particularly true when a worker is exposed to a combination of factors although this varies for different injury types and body locations.^12^

### Attempts to reduce workplace manual handling injuries

Approaches to reduce workplace injuries can be broadly defined into two groups – those that attempt to minimize risk and those that attempt to predict risk.

Approaches directed at minimizing risk include:

1. Legislation and regulation
2.
  - The Queensland Work Health and Safety Act 2011 and Hazardous Manual Tasks Code of Practice 2011 described earlier are examples of legislation and regulation implemented in an effort to prevent and manage workplace musculoskeletal disorders.
3. Manual handling training and assistive devices
4.
  - Despite their widespread use, according to a number of recent reviews^13^–^16^ there is little evidence to support manual handling training and assistive devices, such as back supports and lifting equipment, as an effective control measure for reducing manual handling injuries in the workplace.
5. Conditioning, fitness and health promotion programs
6.
  - Onsite health promotion and exercise programs are typically conducted by allied health professionals. However, the scientific evidence to support their effectiveness in preventing workplace injuries is equivocal.^15^,^17^,^18^
7. Ergonomic redesign and participatory ergonomics
8.
  - A systematic review conducted by Rivilis *et al.*^19^ investigated the effectiveness of participatory ergonomic interventions on workplace health across a variety of industries. Interventions included equipment and workplace redesign, changes in task processes, work organization and teams, policy changes, and equipment maintenance programs. The review identified a moderate level of evidence for reduction of musculoskeletal symptoms and injuries, and a reduction in workers’ compensation claims and days lost as a result of the implementation of participatory ergonomic program recommendations.

Approaches directed at attempting to predict include:

1. Medical history, self-reports, and questionnaires
2.
  - There have been a variety of studies into the predictability of musculoskeletal injuries from previous medical history, self-reports, and questionnaires for workplace injuries. Despite some of these studies showing a predictive relationship between previous medical history and workplace injury,^20^–^22^ in the pre-employment scenario in many jurisdictions, it is unlawful for an employer to base an employment decision on past medical or workers' compensation history. As a result, employers have turned to pre-employment and post-offer assessments and screening.
3. Pre-employment and post-offer assessments and screening
4.
  - Studies assessing the predictability of screening tools such as isometric strength tests,^23^–^29^ cardiovascular fitness,^24^,^26^,^27^,^30^ and isokinetic strength tests^24^,^27^,^28^,^31^,^32^ have had variable results in regards to their predictability of injuries based on assessment criteria. However, as with tests that included dynamic lifting components,^27^,^29^,^32^ the predictability of the tests improves as the occupational physical demands approach or exceed the worker's demonstrated capability.

Whilst limited research is available to support some of these screening practices, they are unfortunately either not practical and/or lawful to use in a pre-employment situation as a method for attempting to predict workers at risk of musculoskeletal injury. As a result, job-specific pre-employment functional assessments (PEFAs) are increasing in popularity as part of a holistic risk management approach for the reduction in musculoskeletal injuries in the workplace.

## Objectives

The objectives of this narrative review are to:

1. present an overview of the state of the evidence for pre-employment functional testing;
2. propose a framework for decisions regarding suitability of assessment formats;
3. discuss the role and potential ethical challenges for the physiotherapist in pre-employment functional testing.

## Major Findings

A PEFA is a series of tests that provide objective information about a worker’s functional capacity in relation to the job for which they are applying. It is an abbreviated version, or short-form, Functional Capacity Evaluation (FCE) with test components and assessment criteria based on the inherent requirements of the job, rather than a worker’s injury or overall capacity profiling, as is the case when used for rehabilitation.

A Functional Capacity Evaluation/Assessment (FCE/FCA) is a series of tasks (mostly standardized and systematic) to determine a worker’s capacity for work. Although variable depending on the purpose and product, they typically include an interview, musculoskeletal assessment, and generic or job-specific or injury-specific testing of functional abilities such as postural and dynamic tolerances and manual handling abilities. They take a whole person approach and so a series of tests are used to assess the complex interactions of functional ability.

Functional Capacity Evaluations are generally conducted by allied health professionals, including physiotherapists, who may, or may not, have had formal training in the assessment methodology. Equipment requirements vary between minimal equipment or workplace based tools and electronic computerized equipment and models. There is a wide range of FCE methodologies ranging from workplace based assessments and informal in-house testing procedures to well-documented proprietary systems with formal training and competency assessments. Examples of proprietary FCEs include the JobFit System PEFA, Isernhagen/Workwell FCE (IWS), Matheson FCE, ErgoScience PWPE, Blankenship FCE, Key FCA, and WorkHab FCE.

In the workplace rehabilitation field, there has been an increased focus of late on the predictability of short-form FCEs. Gross *et al.*^33^ found that the floor-to-waist lift (IWS FCE) provided as much predictive ability as the entire FCE in a population of chronic LBP patients. A follow-up study by Gross *et al.*^34^ demonstrated that where subjects met job demands on three items (floor-to-waist lift, 60-second crouching and 30-minute standing tolerances) they returned to work faster than those who did not. Although more validity research is needed, this evidence for the predictability of return to work of injured workers using short-form FCEs is promising.

One of the obvious advantages of a short-form FCE is the significant reduction in time and cost for administering the test, thereby improving its practicality.^35^ To achieve this without a reduction in safety, reliability or validity is an obvious win-win for all stakeholders. The goal of developing a single job-specific assessment tool with application in both injury prevention and injury management would facilitate reassessment of workers throughout the employment continuum and be a valuable risk management tool for preventing injuries and improving workforce retention and productivity.

### State of the evidence for pre-employment functional testing

Much of the evidence surrounding the validity of functional testing is in the context of the injured worker and prediction of return to work.

Innes and Straker^36^ conducted a review of the validity of 28 work-related assessments. The inclusion criteria were that they were currently in use in Australia, currently commercially available, referred to in publications, and focused predominantly on physical factors related to work. Their methodologies varied, in that some were based on individual self-perception of performance and others reliant on observational skills of the assessor, some were computerized, others need specialized equipment, some specifically assessed lifting whilst others assessed a wider range of physical demands. The authors provided a tabulated description of the levels of validity for each type (face, content, criterion, and construct) considered in their review. Overall, the authors summarized that most have limited evidence of validity, with validity ranging from poor to good across different areas with no instrument demonstrating moderate to good validity across all areas. The conclusion was that more research was needed.

A subsequent literature review of FCEs by Jones and Kumar^37^ identified that the main challenge in assessing the quality of FCEs is that the majority of the tools are proprietary and unless trained in the methodology, researchers do not have access to the procedures and therefore can only review them based on the state of the published knowledge. They also identified that most of the research is done on small groups in rehabilitation settings without access to confidential information or results.

Gouttebarge *et al.*^38^ conducted a more detailed study specifically looking at four ‘popular’ FCEs in The Netherlands with 12 papers meeting the inclusion criteria for methodological quality. They reviewed the literature on the reliability and validity of the Blankenship system (BS), Ergos work simulator (EWS), Ergo-Kit (EK), and Isernhagen work system (IWS), and determined that the predictive validity of IWS was good for injured workers and that concurrent validity of EWS and EK was not demonstrated. There were no validity studies for the BS.

In contrast to the previously mentioned studies studying injured workers, the JobFit System PEFA is a short-form job-specific FCE that is being used to determine the suitability of healthy workers for safe job placement. Reliability studies have demonstrated good to excellent reliability.^39^ Preliminary results from a validity study comparing the PEFA results of 336 healthy mine workers to subsequent injury history records identified a predictive relationship between the PEFA score (as determined by comparing the worker’s capabilities to the job demands) and the risk of a back/trunk/shoulder injury from manual handling (RR 3.6, 95% CI 1.5–8.5).^40^

#### Functional capacity assessment components

The design of a job-specific PEFA typically consists of a physical and musculoskeletal screen, aerobic fitness test, postural tolerances and dynamic activities, and manual handling tasks.

Physical screening is used to identify any conditions such as elevated blood pressure, cardiovascular or respiratory abnormalities, or restricted limb movement, which may prevent the worker from safely participating in the required functional tasks. It can also be used to screen for any current injuries or injuries common to the job for which the participant is applying.^41^ In the past, and unfortunately in many cases still today, trunk mobility and muscle strength are also tested in an effort to predict worker performance. As presented earlier, there are numerous publications^23^–^29^ that refute the inclusion of these tests for this purpose alone. Notwithstanding the scientific evidence, a designer of any physical screening test needs to consider that isolated muscle strength tests are not job-specific and may not be justifiable for job placement decisions under current anti-discrimination legal requirements.

Aerobic physical fitness testing is often included in pre-employment testing as a potential predictor of physical injury. A number of studies have indicated that there is a protective effect of higher aerobic fitness against the incidence and cost of back injuries.^30^,^42^,^43^ In a job-specific pre-employment assessment, fitness tests are included primarily to determine whether the worker has the aerobic capacity to perform the required tasks.

Postural tolerance and dynamic tolerance tests include activities such as reaching forward, squatting, stooping, climbing, walking, and balancing. Again, their inclusion should be based on the job analysis. Procedures for assessing these tasks between different functional testing methods are extremely varied and their reliability depends greatly on standardized procedures for assessment.^36^ Information directly related to these tasks and their predictive value in pre-employment testing was scarce in the peer-reviewed literature. No references to injury predictability of postural tolerance tests specifically in a pre-employment setting were found in a recent Cochrane review.^44^ Much research is needed into the reliability and validity of postural tolerance and dynamic tolerance testing in the pre-employment context.

In contrast, there is a much wider range of published information, and subsequent debate, about the methodology for, and validity of lifting assessments. There are two main topics of debate. Firstly, what comprises safe lifting? Secondly, which is a more accurate predictor of performance – isometric, isokinetic, kinesiophysical, or pyschophysical tests? Kinesiophysical and psychophysical manual handling testing both employ a progressively weighted functional lifting technique. Psychophysical tests allow the participant to determine when their safe maximal lift is reached.^45^,^46^ Both are used in tests of functional capacity with several examples presented in the following paragraphs.

A well-known and widely accepted example of the use of the psychosocial method is by Snook and Ciriello^46^ to determine the Maximum Acceptable Limit Tables. These tables are aimed at assisting industry in the evaluation and design of manual handling tasks for injury prevention, but they are not a prescriptor for the maximum allowable weights that a worker can lift in a functional test, including pre-employment situations.

An interesting study conducted by Chen *et al.*^47^ measured the static lifting strength in a vertical upward lift and toward body lift at various heights for 21 experienced workers and 21 novices. They found that overall, novices lifted 4.5–7.5 kg less than the experienced worker and that their working postures were different. That is, the workers adopted a safer and more skilful technique. This study highlights that studies on students (i.e. novices) may underestimate the abilities of the working population. It also indicates that inexperienced workers may be at a disadvantage compared to experienced workers when conducting manual handling activities in a pre-employment situation, particularly if their work simulation activities use worksite equipment. Whilst static lifting strength was measured in this study, it indicates that if the psychosocial lifting method is used in pre-employment testing situations (particularly for inexperienced applicants), then candidate reports should be supplemented by objective observations of the workers' biomechanical and physiological responses to the lifting activities.

In pre-employment testing, the psychosocial method could be considered potentially unsafe as the participant is likely to be unaffected by injury, potentially unfamiliar with the equipment, and motivated to attempt to exceed their safe demands in an effort to secure employment.

Kinesiophysical testing relies on the ability of the test administrator to observe signs that would indicate that the participant has reached their safe maximal ability, such as increases in heart rate, and deterioration in movement patterns and body mechanics. They also have the advantage of providing the assessor insight regarding the reason for the limitation and therefore provide direction for programs to improve the participant’s abilities.^45^ Smith^48^ used the kinesiophysical approach to assess floor to waist lift in a study of 21 participants with lower back pain. Participants were provided with instruction on the ‘safe’ lifting technique and observing therapists noted when the lift became unsafe. High inter- and intra-rater reliabilities were established between therapists, but it was noted that the determination of safe maximal lift needs more than just observation (e.g. patient reports of discomfort, HR monitoring) as it is a high level judgement by the assessor.

There is, however, also limited evidence to support the validity of the kinesiophysical testing approach.^49^ To maximize safety, a combination of the two is recommended for the physiotherapist assessor, whereby the participant is instructed to cease the task at any time that they do not feel safe even if the assessor has not yet reached that conclusion.

There are a number of notable gaps in the published literature on FCEs. These were regarding studies published on the topics of functional testing in relation to: healthy workers; non-back related injuries; validity studies; pre-employment/post-offer studies; shift work and extended shifts. Even with traditional long-form FCE methods, the consistent conclusion from reviews of the validity of functional testing^36^,^37^,^44^ was that there is minimal quality evidence for functional testing, particularly in healthy workers, despite their increasing use for this purpose.

McHugh and Gibson^50^ highlighted that the limited evidence is not just about validity, but also about the practical application and current practices surrounding the use of PEFAs. Respondents from 29 Australian businesses, mostly employing more than 200 workers, advised that 81% of them matched against the demands of the job but that the majority of the assessments were conducted by medical practitioners including combinations of a general medical exam, practical assessment of physical capacity, and a self-administered questionnaire. These ‘accepted’ inclusions are in contrast to the published literature.

### Framework for assessing suitability of assessment tools

The physiotherapist involved in conducting pre-employment assessments needs to be aware of the availability and strength of the evidence supporting, or rejecting, the inclusion of various components of a job-specific PEFA.

A useful framework for determining the suitability of a chosen assessment tool, ‘Key Attributes of Work-related Assessments’ is presented in Table 1. It is based on the National Institute for Occupational Safety and Health (NIOSH) criteria for the development and selection of work-related assessments, with the key attributes summarized by Innes and Straker^51^ as: safety, reliability, validity, practicality, and utility.

**Table 1:** Key attributes of work-related assessments

Attribute	Description
Safety	The test is safe to administer for both the participant and the assessor
Reliability	The test results are reproducible on any occasion between evaluators (inter-rater) and participants (test-retest)
Validity	The test measures what it reports to measure and is predictive of performance
Practicality	The test is easy to administer with reasonable or minimal cost
Utility	The functional test relates to job performance and meets the needs of the involved parties

It would be reasonable to conclude from the published literature at this time that there is likely to be no single assessment tool that would meet all of these key attributes at the highest level. Instead, it is proposed that the evaluating physiotherapist considers the above attributes and weighs up the available assessment tools based on their order of priority or value of these attributes.

#### Limitations

There are a number of practical limitations of functional testing. The first is that an FCE is an assessment of a participant’s performance at that point in time and it is no guarantee that the worker will be able to, or willing to, perform at the same level on a different occasion in a different location.

A second limitation is regarding the extrapolation of worker performance in the test to a usual workday. Information related to frequency of activity extrapolated from FCEs is traditionally based on an 8-hour day. However, when the duration of an FCE ranges in time from an hour to two days it is obvious that there is no exact formula for projecting occasional, frequent, and constant frequencies to an 8-hour day, 10-hour day, or even an 12-hour day.

A third limitation is differentiating capacity from effort. Pransky and Dempsey^49^ advise that it is difficult to determine what a worker can do versus what they will do. These kinds of statements generally refer to judgement of a worker’s sincerity of effort and so the advantage in a pre-employment situation is that the therapist can assume that the worker is putting forward their best effort and is not distracted by the determination and assessment of sincerity of effort, pain avoidance, and self-limiting behaviours.

A fourth limitation is that FCEs have a particularly narrow focus in their measurement of a worker’s demonstrated abilities and don’t take into account skill, motivation, psychosocial factors^52^ or the physical environment (e.g. temperature, ground, vibration), the psychosocial environment (e.g. production requirements, team dynamics), and their impact on a worker’s projected abilities on the job.

### The role of the physiotherapist and ethical challenges in pre-employment testing

The legal requirement for work-related assessments to be based upon the inherent requirements of the role to meet anti-discrimination requirements and worker safety was outlined in the introductory paragraphs. In summary, it is lawful for an employer to discriminate based on the inherent requirements of the job if employing the worker would pose a threat to themselves or others and their ‘disability’ could not be accommodated without undue hardship to the employer. As recommended by Hoffman and Pransky,^53^ health providers need to have a clear understanding of the legal requirements relevant to their jurisdiction so that they do not provide misleading or inappropriate information that could misinform or potentially put the employer in breach of legislation.

Where the ethical challenge comes into play is largely in the role of the physiotherapist. The physiotherapist’s primary role is typically seen as being responsible to the ‘patient’ (i.e. worker), until it becomes a public health issue. Whilst this is clearer when it comes to infectious diseases, it can also be argued that a worker who is not physically capable of performing the inherent requirements of their role can be placing their co-workers at increased risk of injury as a result of uneven workload due to task selection and rotation, as well as reduced contribution to team-based manual handling activities. There is also the argument that the employer who has requested and is paying for the health professional’s fees is actually the ‘client’ and that the health professional’s responsibility is in protecting them from ‘harm’ – in this case from employing a worker who does not have the capacity to perform the role and who may subsequently be putting themselves and their co-workers at risk of injury resulting in increased workers' compensation costs, reduced productivity, and potential litigation.

Tabershaw^54^ describes the scenario as a shared responsibility. The occupational health professional’s role is in assessing both the risk of the workplace on the worker and the worker on the workplace. To do this well, the physiotherapist needs to not only be competent but also to use appropriate assessment techniques and know about the job. They should not only advise the employer on how to manage and minimize the risks, but they also need to inform the worker regarding the hazards of the job.^54^ The necessity for communication of the workplace hazards to the worker is also echoed by the legal fraternity. Guthrie and Westaway^55^ advise that employers should disclose the job requirements to the workers so that they can make their own decisions regarding their capacity to do that job. A PEFA is therefore an opportunity for a worker to ‘experience’ the physical demands of the job for which they are applying and leverage their subsequent determination of self-efficacy with feedback from the assessment to make an informed decision about their perceived ability to do the job and to determine if there is an acceptable level of risk to them.

Much of the focus in the published literature and industry discussions is on the ‘cost’ of workplace injury. An area that warrants further discussion within our industry is the financial, emotional, and social ‘cost’ of not employing a worker based on the results of a PEFA.

A final topic that has not been discussed in the published literature, yet is frequently raised in informal professional interactions, is that of ‘acceptable loads’ during pre-employment testing. Whilst the discussion points raised above clearly point to the necessity for job-specific assessments based on actual job demands, the physiotherapist is often at an ethical crossroads, when in their opinion, or based on normative data, these job demands result in ‘unacceptably high’ manual handling requirements. The health professional and worker can be confident, and comforted, knowing that either of them can stop the assessment at any time that they feel the activity becomes unsafe. However, in the event that the physiotherapist chooses to artificially limit the assessment based on their personal opinion of what constitutes an ‘acceptable weight’ and that in turns limits the worker’s ability to demonstrate their full capacity, then that worker may be unfairly disadvantaged in the recruitment and selection process. Conversely, if the employer and therapist artificially lower the job demands, then they are likely to produce a number of false positive results, which could result in insufficient risk management strategies being employed. This reinforces the physiotherapist’s multiple responsibilities to: follow consistent assessment procedures; observe safe lifting practices; and work with the employer to reduce the job demands. Future research into the comparison between health professionals’, employers’, and workers’ attitudes and beliefs on this ethical dilemma would make for an interesting discussion.

## Conclusion

Pre-employment functional assessments have a number of uses. They can be used to: identify a worker’s current injuries or restrictions in movement for appropriate management; determine a worker’s ability to safely meet the inherent requirements of the job for appropriate job placement; establish a worker’s baseline for health monitoring and to identify the value of wellness and conditioning programs and the effectiveness of rehabilitation programs.

Whilst it is not the physiotherapist’s responsibility to make decision about what risks are acceptable, they are responsible for providing reliable, valid, and meaningful information on which the employer and worker can base those decisions. It is therefore the physiotherapist’s responsibility to adopt ethical and evidence-based practices and choose assessment methods that meet the attributes of excellence for work-related assessments.^51^,^54^ This sentiment is echoed by many in the field^44^,^56^ as they discuss the need for further research on the validity and development of evidence-based guidelines for functional testing in healthy workers.

Just as physiotherapists are but one discipline involved in the prevention of workplace musculoskeletal injury, PEFAs are but one part of a holistic injury prevention program including ergonomics, behavioural safety, physical conditioning, and education.

## Disclosure statement

This narrative review is based on the literature review prepared by the author for the degree of Doctor of Philosophy at The University of Queensland, Australia. The author is a Director of JobFit Systems International that utilizes the JobFit System PEFA in its commercial operations.
